# Chemically Induced Photoswitching of Fluorescent Probes—A General Concept for Super-Resolution Microscopy

**DOI:** 10.3390/molecules16043106

**Published:** 2011-04-13

**Authors:** Ulrike Endesfelder, Sebastian Malkusch, Benjamin Flottmann, Justine Mondry, Piotr Liguzinski, Peter J. Verveer, Mike Heilemann

**Affiliations:** 1 Biotechnology & Biophysics, Julius-Maximilians-University Würzburg, Am Hubland, 97074 Würzburg, Germany; 2 Bioquant Centre, University of Heidelberg, INF 297, 69120 Heidelberg, Germany; 3 Department of Systemic Cell Biology, Max Planck Institute of Molecular Physiology, Otto-Hahn-Strasse 11, 44227 Dortmund, Germany

**Keywords:** photoswitchable organic fluorophores, fluorescent proteins, super-resolution, PALM, dSTORM

## Abstract

We review fluorescent probes that can be photoswitched or photoactivated and are suited for single-molecule localization based super-resolution microscopy. We exploit the underlying photochemical mechanisms that allow photoswitching of many synthetic organic fluorophores in the presence of reducing agents, and study the impact of these on the photoswitching properties of various photoactivatable or photoconvertible fluorescent proteins. We have identified mEos2 as a fluorescent protein that exhibits reversible photoswitching under various imaging buffer conditions and present strategies to characterize reversible photoswitching. Finally, we discuss opportunities to combine fluorescent proteins with organic fluorophores for dual-color photoswitching microscopy.

## 1. Introduction

**Figure 1:**
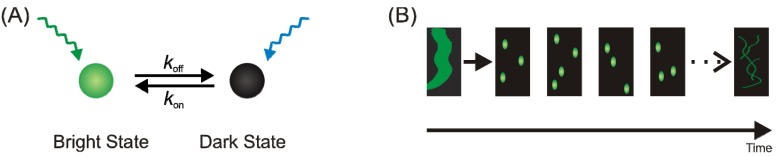
The principle of localization-based super-resolution microscopy with photoswitchable fluorophores. **(a)** Photoswitchable fluorescent probes exist in a bright (fluorescent) and dark (non-fluorescent) state, and can be interconverted e.g. by irradiation with light. **(b)** The total fluorescence signal is separated in time by activating a sparse subset of fluorophores. Single-molecule coordinates are determined and used to reconstruct a super-resolution image.

The limited resolution of light microscopy has lead to the development of a number of technologies that are referred to as super-resolution microscopy [1,2]. The most prominent technologies in this toolbox are stimulated-emission depletion (STED) [3], structured-illumination microscopy (SIM) [4] and single-molecule localization based methods [5,6,7]. Among the different methods, only the single-molecule localization-based techniques have demonstrated near-molecular resolution in biological samples. Prominent examples for these techniques are photoactivated-localization microscopy (PALM) [6], stochastic optical reconstruction microscopy (STORM) [5] and direct STORM (*d*STORM) [7]. The working principle of all these techniques is a temporal confinement of the fluorescence signal by employing photoswitchable or photoactivatable fluorescent probes, single-molecule localization with high precision and image reconstruction (Figure 1).

The key requirement of all localization-based super-resolution technologies is the availability of fluorescent probes that can be photoactivated or photoswitched [8]. Different concepts for localization microscopy have been introduced. STORM applies carbocyanine fluorophores such as Alexa647 or Cy5 and an additional activator fluorophore [5]. However, photoswitching of carbocyanine fluorophores requires the removal of oxygen [5,9] and is not compatible with live cell imaging. PALM applies photoactivatable or photoconvertible fluorescent proteins [6], has the advantage of genetic co-expression and thus stoichiometric labeling of a target protein, and also can be combined with live cell imaging [10]. The more general *d*STORM approach operates a broad range of synthetic organic fluorophores as photoswitchable units [7,8,11], and in combination with site-specific tagging approaches, can be used inside living cells [12]. A selection of photoswitchable and photoactivatable fluorophores that are nowadays used in the various approaches of super-resolution localization microscopy is presented in Table 1.

In the present paper, we first introduce the mechanisms of photoswitching of organic fluorophores under reducing conditions as they are used in the *d*STORM principle [7,11,13,14]. Building on these findings, we present our studies on how photoactivatable and photoconvertible fluorescent proteins perform under similar imaging conditions as in *d*STORM, which is under reducing buffer conditions and both in the presence and absence of oxygen. Finally, we discuss possible experimental conditions that allow the combined use of both fluorescent proteins and organic fluorophores in dual-color localization-based super-resolution imaging with photoswitchable probes.

**Table 1:** A selection of fluorescent proteins and organic fluorophores that can be photoactivated or photoswitched and that are used in super-resolution localization microscopy (rev. = reversible, irrev. = irreversible) [7,11,13,15,16,17,18,19,20,21].

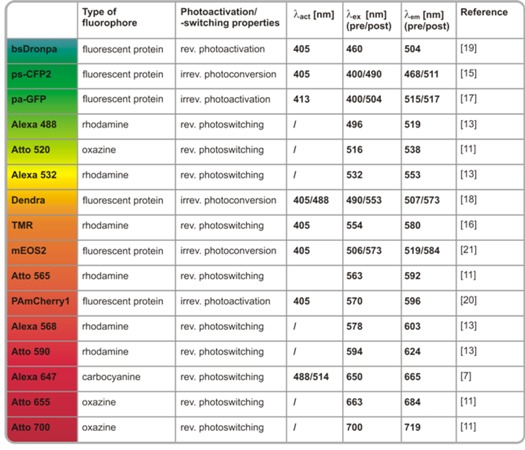

## 2. Results and Discussion

### 2.1. Operating organic fluorophores as photoswitches using redox chemistry

Synthetic organic fluorophores can be operated as photoswitchable fluorophores by exploiting their sensitivity towards reducing agents, e.g. thiol compounds, which lead to fluorescence quenching through the formation of a stable non-fluorescent state [13,14]. For the application in single-molecule localization based super-resolution imaging, a suitable fluorophore should in addition exhibit a high brightness, such that the detection of single molecules is possible. Synthetic organic fluorophores that have successfully been used for localization-based super-resolution imaging using the *d*STORM approach are e.g. the carbocyanine fluorophores (Cy5, Alexa Fluor 647 and others) [5,9], rhodamine fluorophores (Alexa Fluor 488, Alexa Fluor 532 and others) and oxazine fluorophores (e.g. ATTO655) [8,14].

**Figure 2:**
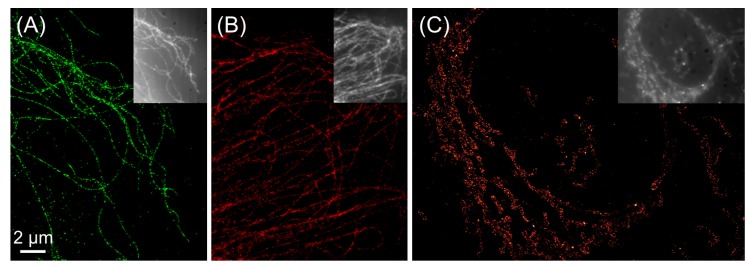
*d*STORM imaging operating conventional synthetic organic fluorophores as photoswitches through redox-chemical processes: (a) microtubulin labeled with Alexa Fluor 532, (b) microtubulin labeled with ATTO655, (c) endoplasmatic reticulum labeled with Alexa Fluor 647.

*d*STORM imaging of samples is straightforward as it can be combined with established techniques such as immunofluorescence using commercially available antibodies that carry a suitable fluorophore that can be operated as a photoswitch. *d*STORM imaging of carbocyanine fluorophores such as Alexa Fluor 647 or Cy5 is performed using an imaging buffer suited for carbocyanine photoswitching and simultaneous irradiation with 514 nm and 647 nm [5,9]. Rhodamine fluorophores, such as Alexa Fluor 532 and others, follow a slightly different mechanism. These fluorophores are also photoswitched in the presence of millimolar concentrations of MEA, but compared to the carbocyanine fluorophores tolerate oxygen. The stable off state of rhodamine fluorophores has been demonstrated to be a radical ion [13,14]. In a similar way as rhodamines, oxazine fluorophores such as ATTO655 can be operated as photoswitches in the presence of oxygen and by adding millimolar concentrations of MEA. The photoproducts in photoswitching of oxazine fluorophores have been studied with electron paramagnetic resonance (EPR) spectroscopy and by Fourier-transform infrared (FT-IR) spectroscopy in combination with theoretical calculations and involve both a radical ion as well as the transition to a fully reduced state [13,14,22]. Exemplary super-resolution images for one representative fluorophore of each of these three classes have been recorded with the *d*STORM approach and are shown in Figure 2. Beyond super-resolution imaging in fixed cells, some organic fluorophores can be operated as photoswitches inside living cells, where fluorescence quenching is caused by intracellular thiols or reducing species like glutathione [12,16]. The necessary site-specific labeling of proteins with organic fluorophores can be realized with the various chemical tags available, such as the SNAP-Tag [23] or trimethoprim tag [12].

### 2.2. Photoswitching of photoactivatable and photoconvertible fluorescent proteins using redox chemistry

Compared to organic fluorophores, photoswitchable fluorescent proteins have the advantage that they can be genetically attached to a protein of interest and provide stoichiometric labeling. In addition, fluorescent proteins do not require specific imaging buffers and can be used for live-cell PALM imaging in a straightforward manner [10]. A slight disadvantage of fluorescent proteins is their lower brightness or photon yield, which directly translates into the localization accuracy and thus the achievable optical resolution [24]. In addition, experiments that aim to follow dynamic processes require multiple read-out cycles of photoswitchable fluorophores [12,25], and only few photoswitchable fluorescent proteins are available for this purpose so far [26]. Bright and at the same time reversibly photoswitchable fluorescent proteins are thus rare, and organic fluorophores are sometimes an attractive alternative choice. Beyond these small differences and next to the particular advantages and disadvantages that both organic fluorophores and fluorescent proteins exhibit, it would be desirable to combine their use in two-color super-resolution imaging.

**Figure 3:**
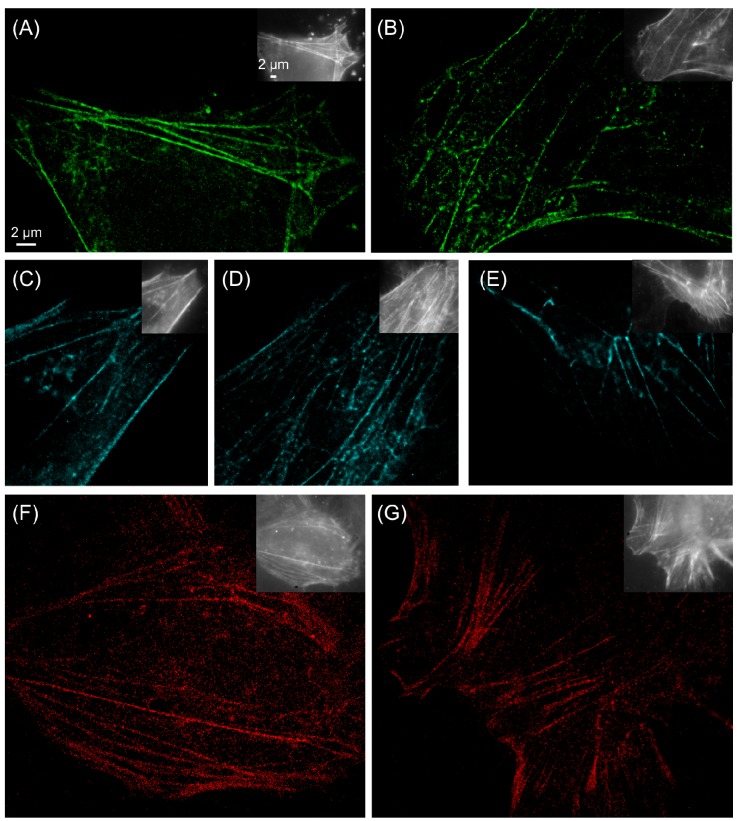
PALM imaging of the actin cytoskeleton in HeLa cells. The photoswitchable fluorescent protein Dendra2 (a, b), the reversibly photoswitchable protein bsDronpa (c-e) and the photoactivatable protein PAmCherry1 (f, g) are shown. Images were recorded in PBS buffer (a, c, f), in the presence of 10 mM MEA (b, d, g) and in the presence of 100 mM MEA (e).

Here, we have studied the photoswitching properties of fluorescent proteins under reducing conditions as they are used for *d*STORM imaging. Therefore, we have compared the photoswitching properties in physiological buffer (PBS), in addition of a reducing agent MEA and in the presence or absence of oxygen. The fundamental initial interest here is to find out whether and how reducing conditions impact the photoswitching properties of fluorescent proteins *per se*. Beyond this, our motivation is three-fold: first, a combination of a suitable organic dye and fluorescent protein would allow dual-color imaging using both organic fluorophores and fluorescent proteins; second, it would allow using complementary labeling strategies; and third, it could present an attractive route for two-color live-cell super-resolution imaging. We have focused in this study onto a selection of five fluorescent proteins previously used for PALM imaging, which are psCFP2 [15], bsDronpa [19], mEos2 [21], PAmCherry1 [20] and Dendra2 [18], and reducing buffer conditions as they are used for *d*STORM imaging [7,13].

**Figure 4:**
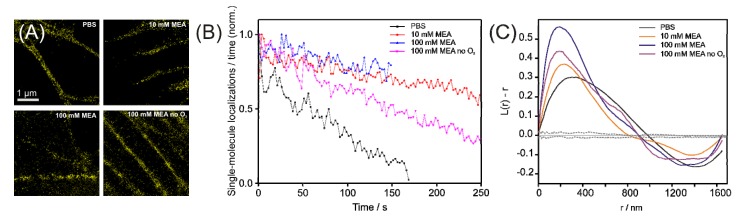
Reversible photoswitching of the fluorescent protein mEos2 under reductive and oxygen-free conditions. (a) PALM images of actin-mEos2 in PBS (HeLa), 10 mM (U2OS), 100 mM MEA (HeLa) and 100 mM MEA with oxygen removed (U2OS). (b) The decay of the relative number of localizations per frame indicates an increased reversible photoswitching of mEos2 in buffers with reducing thiols. (c) Ripley’s k-function corroborates reversible switching by indicating a shift to smaller distances between single mEos2 proteins (black line, expectation value, dotted grey lines, 95% confidence level of the expectation value).

We have performed PALM imaging of cells co-expressing one of the fluorescent proteins with actin monomers. We used various imaging conditions for PALM imaging, i.e. in physiological buffer (PBS pH 7.4), under reducing conditions in the presence of 10 mM MEA, by adding 100 mM MEA, and in 100 mM MEA with oxygen removed from the buffer using an enzymatic oxygen scavenger. We found that all photoswitchable fluorescent proteins can be operated both in PBS as well as in PBS with 10 mM MEA added (Figure 3). This finding suggests that any of the fluorescent proteins can be combined with rhodamine and oxazine fluorophores, as these fluorophores can be operated under similar conditions, e.g. ATTO655 [13,14]. Typically, the addition of higher concentrations of MEA did deteriorate the photoswitching capabilities of the fluorescent proteins. The fluorescent proteins bsDronpa (Figure 3e), psCFP2, Dendra2 and mEos2 (Figure 4a) could be operated as photoswitches in 100 mM MEA, i.e. the highest concentration of MEA tested that is required for photoswitching of many rhodamine fluorophores [14]. Both psCFP2 (Supporting Figure 1) and mEos2 also tolerate oxygen removal and thus image buffer conditions as required to photoswitch carbocyanine fluorophores, e.g. Alexa Fluor 647, Alexa Fluor 680, Cy5 and Cy5.5 [7].

The photoswitching properties in different buffer conditions, *i.e.* PBS only, PBS with 10 mM MEA and PBS with 100 mM MEA and oxygen removed, were characterized in more detail for the fluorescent protein mEos2, as this protein both tolerated all buffer conditions and exhibited the highest photon yield (Figure 4a). For this purpose, two different approaches were used. In a first approach, the number of single-molecule localization was plotted against time for the different imaging buffer conditions (Figure 4b), while the experimental parameters such as excitation intensities were kept constant. We observe an increase in the number of single-molecule localizations over time in the presence of reducing agents as well as in the presence and absence of oxygen. This finding suggests an increase in reversible photoswitching of mEos2 in the photoconverted form and is in accordance with previous findings [27]. As an alternative approach, we computed Ripley’s k-function [28] in order to characterize reversible photoswitching or apparent clustering through an all distance distribution in regions of globular actin monomers (Figure 4c) (for details see experimental section). We observe a shift of the main maximum of Ripley’s k-function towards shorter r-values in reducing and oxygen-free buffer. Given that the underlying distributions of fluorescent proteins used to compute the k-function was similar for all four data sets, we interpret this finding as increase in reversibility of photoswitching of mEos2 in the photoconverted form. Beyond the shift in the maximum, we observe a more confined shape of the k-function in the presence of reducing thiols as well as in the absence of oxygen. As the k-function essentially reports on single-molecule distributions over a range of distances [28,29], we interpret this confinement in an increased contribution of multiple single-molecule localizations through reversible switching of the same fluorescent protein.

### 2.3. Prospectives for dual-color localization-based super-resolution imaging

**Figure 5:**
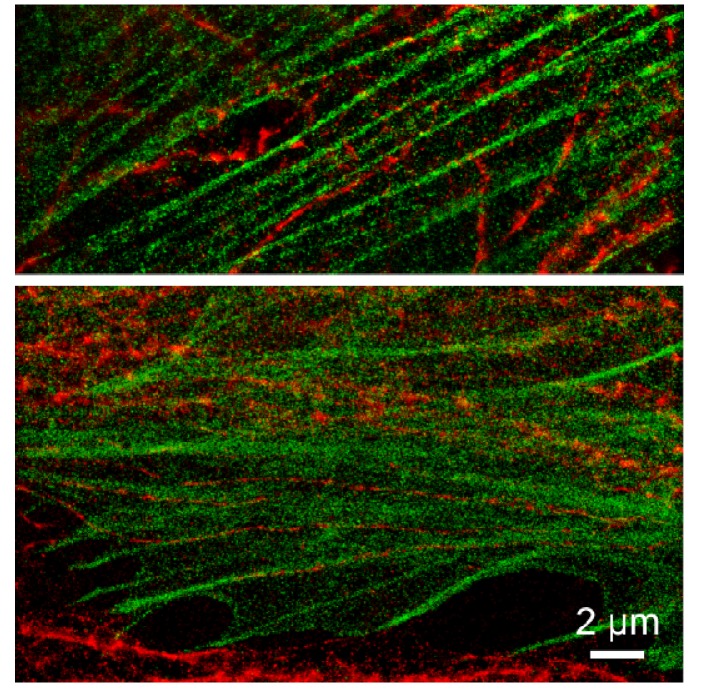
Dual-color imaging of HeLa cells with mEos2-actin (green color) and ß-tubulin labeled with ATTO655 (red) via immunofluorescence in 10 mM MEA.

The photoswitching properties of the fluorescent proteins in the different imaging conditions open a number of possibilities to combine photoswitchable fluorescent proteins with photoswitchable organic fluorophores (summarized in Table 2). Essentially, all five proteins can be operated in combination with rhodamine and oxazine fluorophores in physiological buffer and in the presence of 10 or 100 mM MEA (see Table 2) (Figure 5). In addition, the fluorescent protein mEos2 can even be operated under buffer conditions as used for the carbocyanine fluorophores Alexa Fluor 647 and Cy5.

**Table 2:** Summary of suitable buffer conditions for the fluorescent proteins tested and with respect to organic fluorophore photoswitching.

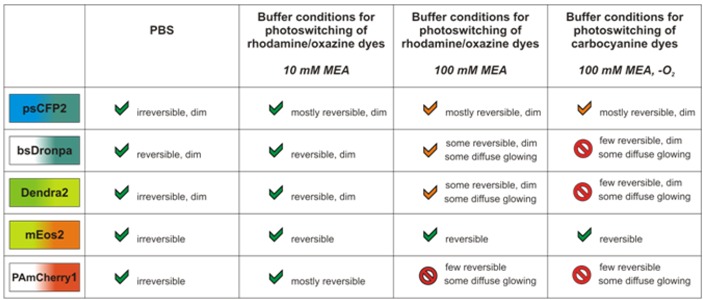

## 3. Experimental

### 3.1. Sample preparation

HeLa or U2OS cells were plated and grown in Labtek 8-well chambered cover glass (Nunc, Germany) up to 80% confluency. The cells were transiently transfected with a plasmid encoding for one of the fluorescent proteins (mEos2, PAmCherry1, bsDronpa, psCFP2, Dendra2) and actin using the transfection reagent Turbofect (Fermentas, Germany). After overnight expression, the cells were fixed using 4% PFA for 10 minutes and stored in phosphate buffered saline (PBS). Labeling with organic fluorophores was done using standard immunofluorescence protocols. The microtubulin network of COS-7 cells was labeled using a rabbit anti-β-tubulin primary antibody (Abcam, Cambridge, UK). The secondary antibody was a goat F(ab’)_2_ anti rabbit (Invitrogen, USA) that was custom-labeled with ATTO 655 following protocols published by the manufacturer (Atto-Tec, Germany). The degree of labeling was determined to 1.3. Microtubulin of HeLa cells was labeled with monoclonal anti-α-tubulin antibody (MS-581-P1; Thermo Scientific, Waltham, MA) and Alexa Fluor 532 labeled secondary antibody (A11002, Invitrogen, Carlsbad, CA). Staining of the endoplasmatic reticulum of HeLa cells was done using monoclonal antibody against calnexin (ab31290; Abcam, Cambridge, UK) and Alexa Fluor 647 labeled secondary antibody (A21235; Invitrogen, USA).

### 3.2. Microscopic configuration

A standard epifluorescence microscope operated in total internal reflection fluorescence (TIRF) and equipped with suitable laser sources was used for PALM and *d*STORM imaging, essentially configured as published elsewhere [6,7,8]. Briefly, a multi-line argon-krypton laser (Innova 70C; Coherent, USA) was coupled into an inverted microscope (IX71, Olympus, Japan) equipped with a 60x oil immersion objective (PlanApo 60x, NA 1.45, Olympus). Excitation and fluorescent light was separated using a dichroic mirror. Here either FF560/659-Di01 (AHF, Germany) or FF410/504/588/669-Di01 (AHF, Germany) or HC576/661 (AHF, Germany) were used. The fluorescence signal was read out with an electron-multiplying CCD camera (EMCCD; Andor Ixon DU 897, Belfast, Ireland). Raw data was processed using the rapidSTORM algorithm [30].

### 3.3. PALM imaging

Fluorescent proteins were imaged with a suitable combination wavelengths provided by various laser sources: 378 nm or 405 nm and 488 nm (psCFP2, bsDronpa), 378 nm or 405 nm and 568 nm (mEos2, PAmCherry1, Dendra2). Typically, irradiation intensities of 20 to 40 mW (1–2 kW cm^-2^) for the read-out wavelength (488 nm or 568 nm) were used. A lower intensity of about 10 mW was used for imaging bsDronpa. Filter sets for imaging mEos2, PAmCherry1 and Dendra2 included a 568/10 bandpass filter (AHF, Germany) as clean-up filter in the excitation path, and a combination of a 568LP and a 610/75 (AHF) in the detection path. Filter sets for imaging psCFP2 and bsDronpa included a 488/10 clean-up filter (AHF) in the excitation path, and a combination of a 488LP and a 550/88 (AHF) in the detection path. Typically, 3000–6000 frames were recorded at frame rates of 100 Hz, and the experiment was terminated when all fluorescent proteins were irreversibly photobleached. In experiments that showed reversible photoswitching of the fluorescent proteins, a similar number of frames was recorded and the experiment finished prior to all fluorescent proteins photobleached.

### 3.4. dSTORM imaging

*d*STORM imaging was performed in suitable imaging buffers as described elsewhere for carbocyanine [7] and rhodamine or oxazine fluorophores [8]. Briefly, photoswitching of the carbocyanine Alexa 647 was performed with 514 nm and 647 nm radiation in oxygen-depleted PBS buffer and 100 mM MEA added. Photoswitching of ATTO655 was performed in 10 mM MEA and with irradiation at 647 nm. Photoswitching of Alexa Fluor 532 was performed in 100 mM MEA and with irradiation at 514 nm.

### 3.5. Dual color imaging

Dual color imaging of ATTO655 and mEos2 was performed by sequentially imaging first ATTO655 and then mEos2. As imaging buffer, 250 mM NaHCO_3_ (pH8) with 10 mM MEA was used. The filter sets used were a 488/568/647-10 bandpass filter (AHF, Germany) for the excitation path. In the detection paths, a combination of a LP647 and 705/75(AHF) (ATTO655 channel) and a combination of LP568 SP630 and 610/75 (AHF) (mEos2 channel) was used. The two channels were aligned by imaging multi-labeled beads (TetraSpeck, Invritrogen) and calculating the local weighted mean using the local weighted mean function of Matlab (Mathworks, Natick, MA) [31].

### 3.6. Data analysis

Reversible switching was assessed through calculating the distribution of all localization distances within the population of distinct regions of interest (ROI) with the size of 1.65 × 1.65 µm². In order to have comparable input data, we chose intracellular regions with a high occurrence of globular actin as ROI. Regions with filaments were avoided, because of a variable and dense structure that is typical for actin multimers. The spatial distribution of all localizations was calculated by Ripley’s K-function [28,29]:

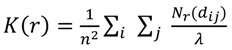
(1)
where n is the total number of localizations within the ROI, dij is the distance between the localization i and j, Nr is the number of localization around localization i within the distance j and λ is a weighting factor correcting for the area of the ROI.

The function is linearized:

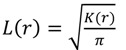
(2)
and then normalized:

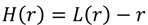
(3)
so that a normal distribution would give an expected value of H(r) = 0 for all r [32].

In order to account for edge effects we chose the torroidal edge correction [33]. To achieve statistical significance we employed 95% confidence envelopes produced with Monte Carlo methods [32].

## 4. Conclusions

We have studied the photoswitching properties of the photoactivatable fluorescent protein PAmCherry1, of the photoconvertible fluorescent protein mEos2, Dendra2 and psCFP2 and of the photoswitchable fluorescent protein bsDronpa. We found that all five fluorescent proteins tolerate both physiological buffer conditions (PBS) as well as the addition of a reducing agent MEA at concentrations of 10 mM. Furthermore, we found that all proteins except PAmCherry1 tolerate higher concentrations of 100 mM MEA, as they are required also in photoswitching of some rhodamine fluorophores. Finally, we found that mEos2 and psCFP2 tolerate imaging conditions as used for photoswitching carbocyanine fluorophores, *i.e.* 100 mM MEA and an oxygen-free buffer. We have analyzed the impact of the various buffer conditions on the photoswitching properties for the fluorescent protein mEos2 in more detail using single-molecule localization data, and we identified reversible photoswitching. Overall, this study suggests the combination of fluorescent proteins and organic fluorophores in single-molecule based super-resolution imaging, both in fixed and in live cells.

## Supplementary Materials

Supplementary Materials includes PALM images of actin-psCFP2 and can be accessed at http://www.mdpi.com/1420-3049/16/4/3106/s1.
