# Supplementary material for: Chemically Induced Photoswitching of Fluorescent Probes—A General Concept for Super-Resolution Microscopy
Source: Molecules. 2011 Apr 13;16(4):3106–18. doi: 10.3390/molecules16043106 (PMC6260607; doi:10.3390/molecules16043106)

Supplemental Information

Additional super-resolution images recorded with the PALM technique of actin-psCFP2 (Supporting Figure 1) are shown.

Supporting Figure 1. PALM images of actin-psCFP2 recorded in (A) PBS, (B) PBS with 10 mM MEA, (C) PBS with 100 mM MEA and (D) PBS with 100 mM MEA and oxygen removed.

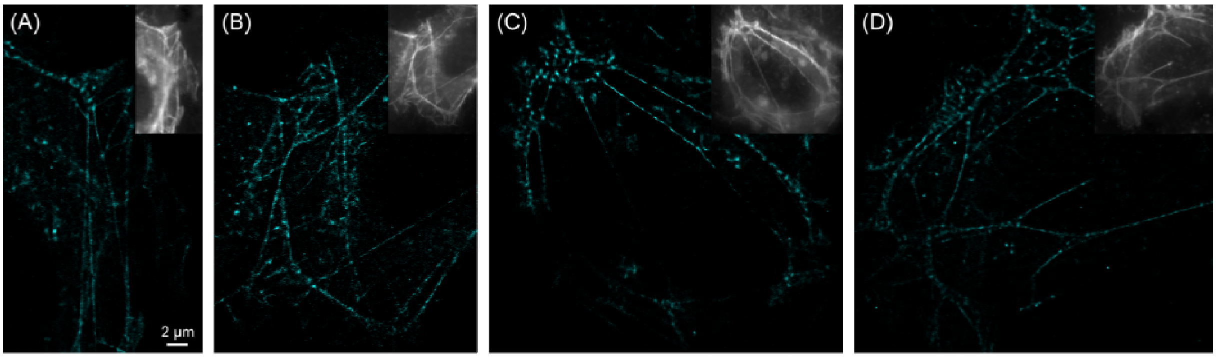

Supplement: Supplementary file 1 [file molecules-16-03106-s001.pdf]
